# Genetic Population Structure of Cacao Plantings within a Young Production Area in Nicaragua

**DOI:** 10.1371/journal.pone.0016056

**Published:** 2011-01-14

**Authors:** Bodo Trognitz, Xavier Scheldeman, Karin Hansel-Hohl, Aldo Kuant, Hans Grebe, Michael Hermann

**Affiliations:** 1 Austrian Institute of Technology, Seibersdorf, Austria; 2 Bioversity International, Cali, Colombia; 3 Pro Mundo Humano, Managua, Nicaragua; 4 Crops for the Future, Serdang, Selangor, Malaysia; Aarhus University, Denmark

## Abstract

Significant cocoa production in the municipality of Waslala, Nicaragua, began in 1961. Since the 1980s, its economic importance to rural smallholders increased, and the region now contributes more than 50% of national cocoa bean production. This research aimed to assist local farmers to develop production of high-value cocoa based on optimal use of cacao biodiversity. Using microsatellite markers, the allelic composition and genetic structure of cacao was assessed from 44 representative plantings and two unmanaged trees. The population at Waslala consists of only three putative founder genotype spectra (lineages). Two (B and R) were introduced during the past 50 years and occur in >95% of all trees sampled, indicating high rates of outcrossing. Based on intermediate allelic diversity, there was large farm-to-farm multilocus genotypic variation. GIS analysis revealed unequal distribution of the genotype spectra, with R being frequent within a 2 km corridor along roads, and B at more remote sites with lower precipitation. The third lineage, Y, was detected in the two forest trees. For explaining the spatial stratification of the genotype spectra, both human intervention and a combination of management and selection driven by environmental conditions, appear responsible. Genotypes of individual trees were highly diverse across plantings, thus enabling selection for farm-specific qualities. On-farm populations can currently be most clearly recognized by the degree of the contribution of the three genotype spectra. Of two possible strategies for future development of cacao in Waslala, i.e. introducing more unrelated germplasm, or working with existing on-site diversity, the latter seems most appropriate. Superior genotypes could be selected by their specific composite genotype spectra as soon as associations with desired quality traits are established, and clonally multiplied. The two Y trees from the forest share a single multilocus genotype, possibly representing the Mayan, ‘ancient Criollo’ cacao.

## Introduction

In Central America, the cacao tree (*Theobroma cacao* L.), a plant of the humid neotropics, was already being cultivated by the Olmecs and early Mayas, 3000 years ago. Recent investigations on the origin of the ancient Central American cacao, traditionally referred to by its morphogeographic name ‘Criollo’, suggest that it may have been introduced from an area now in Venezuela, adjacent to the center of highest diversity of *Theobroma cacao* L. in upper Amazonia [1]. However, Criollo cacao represents only a small part of the allelic bandwidth of cultivated and natural cacao populations occurring in Amazonian forests where the species originated. Today's descendants of the Mayan ancient Criollo cacao can therefore be considered as the products of multigenerational selection by Amerindian farmers [2], [3]. Hybrids of Criollo and some Forastero accessions, known as Trinitario or modern Criollo [2], [4], and as ‘Trinidad Selected Hybrids’ (TSH), are renowned for their distinct aroma making them a preferred raw material for fine cocoa chocolate [5]. Therefore, remaining sources of ancient Criollo that can still be found in Central America, including Nicaragua, contain potentially valuable germplasm for future breeding of high quality cacao.

Types of cacao are distinguished by several partly overlapping naming schemes. There is the traditional recognition of morphogeographical groups or cultivars (Criollo from Central America, Forastero from Amazonian South America, Amelonado, a Forastero with distinct fruit shape, Trinitario from Trinidad and Tobago, and Refractario from Ecuador originally selected for its resistance to witches' broom disease, *Crinipellis perniciosa* (see also http://sta.uwi.edu/cru/icgt/types.asp). Traditional traders' ‘varieties’ are recognized by the trade quality (e.g., Trinitario, Criollo, Amelonado, Catongo, Nacional) [4], and cocoa and chocolate are frequently graded and marketed under the name of the country (or region) of production, e.g. Amazonia, Belize, Ecuador, Ivory Coast, or Venezuela. Although modern plantations are often composed of clones (grafted trees or rooted cuttings), propagation by seed has been the simple traditional method for the multiplication of cacao trees. Cacao possesses poorly characterized sexual self-incompatibility, but many trees under cultivation are sufficiently self-fertile [6] to allow for secure yields, and to give rise to inbreeding. The use of clonally propagated, bred and selected cultivars, as are widely used with many horticultural fruit crops in temperate zones, is only just beginning.

The gourmet chocolate sector makes up 4% of the total world chocolate market (S. Vervliet, Puratos/Belcolade, 2007, pers. comm.) but is growing quickly. ‘Fine-flavor cocoa’ fetches a considerable price premium, up to four times of the price of standard commodity cocoa. The manufacture of gourmet chocolate depends to a large extent on intrinsic cocoa qualities which are determined by genotype, and on-farm processing including the selection of pods, fermentation, and drying of beans. This offers good opportunities for quality differentiation and value addition that would benefit the growers (S. Petchers, CATIE, 2004, pers. comm.). However, cacao is predominantly produced by smallholder farmers whose level of training and organization in the production chain is often insufficient to maximize the benefits from the production of high quality cocoa.

In Nicaragua, one of the largest cocoa production areas is found in the municipalities of Waslala and Rancho Grande, towns in the central northern part of the country. In pre-Colombian times, this area was under cultural and linguistic influences from the Mayas, and from the Aztecs further north [7]. Waslala is equidistant between the Pacific and Atlantic oceans, at an elevation of 200–740 m in a south-east facing depression adjacent to the Peñas Blancas massif. The average annual temperature ranges between 21.3 and 24.9°C, and mean annual rainfall is between 2170 and 2660 mm (Worldclim database, www.worldclim.org [8]). The beginnings of commercial cocoa cultivation date back to 1961 (E. Rios, first president of the cocoa producers cooperative Cacaonica, 2007, pers. comm.). During and after the civil war in the 1980s, refugees and migrants from all over Nicaragua arrived, and cocoa production has greatly expanded since 1991 with the establishment of the non-governmental organization Pro Mundo Humano, and the foundation of Cacaonica. Cocoa has since become a popular cash crop. The area planted with cacao is now some 1700 ha, having increased during the past five years due to the attractive prices. Typically a household cultivates 0.7–1 ha of cacao, containing 300–600 trees. Plot sizes rarely exceed 2 ha because cacao cultivation is labor intensive, in particular pruning, manual removal of diseased fruits, and continual harvesting and processing. Plantings are distributed on steep slopes that are not useful for cattle pasture. Individual farms rarely yield more than 0.5 t/ha of dried cocoa beans per year, but together, the municipality's total annual crop contributes considerably to the national cocoa production of 2650 t (in 2009). Farmers can obtain higher prices for high quality cocoa grades, especially if organically certified. There is also potential for adding value from quality differentiation based on characteristics imparted through locality-dependent (environmental), management, and genetic factors. Several commercial cocoa and chocolate companies source their raw material in Waslala, including Ritter (Germany), Cocoa S.A. (Costa Rica), Daarnhouver (the Netherlands), and Zotter (Austria).

It is believed that only a limited number of introductions contributed to the present-day germplasm in Waslala cocoa plantings, although few records are available. The Tropical Agricultural Research and Education Center (CATIE, Turrialba, Costa Rica) distributed seed (beans) in the 1980s to Central American countries including Nicaragua (W. Phillips, CATIE, 2007, pers. comm.), some of which arrived at Waslala. In addition, several farmers interviewed during this research claimed to have occasionally brought in seed and scions from other regions, and others reported finding rare pre-existing cacao trees when they arrived at their new farmland in the 1970s and 1980s. Cacao has been predominantly introduced to Waslala as seedlings, and to a lesser extent through grafted clones. The farmers themselves propagate cacao mostly through the use of seedlings.

This paper explores the genetic composition and structure of cacao populations, as a prerequisite for varietal certification and denomination of Waslala cocoa. It also assesses optimal means to improve cocoa yield and quality in this area for the benefit of the farmers and cocoa producers. For the cacao research community, it is of interest to understand how cacao populations are shaped by germplasm introductions and management. We have representatively sampled the municipality and surveyed the genotype of trees by a number of well-defined simple sequence repeat (SSR) markers. It addressed questions related to the extent of allelic diversity and the possibility of discerning the genetic structure of population, with the objective of identifying specific genetic backgrounds which can be related to geographic areas, farmers' degree of access to germplasm, and specific environmental conditions.

## Results

### Descriptive statistics and genetic diversity

The 15 microsatellite primer pairs detected 116 individual alleles (with 7.73 alleles per locus on average) across all samples collected in Waslala municipality. There were no null-alleles apparent. When only one allele was detected, the individual was considered homozygous at this locus. Two trees had three individual alleles at only two SSR loci for unknown reasons. For the analyses of population genetics, the rarer alleles, relative to the entire data set, were considered in these exceptional cases. Six groups of trees sharing an identical multilocus genotype were found, and two of these genotypes were frequent (Table 1, group E with 10 members, and group D with 7 members).

**Table 1:** Trees with matching multilocus genotypes across 15 SSR markers.

Sample	Farm/Location	No. Matches	Label
W042	FBBSB	2	A
W041	FBBSB		A
W356	FJM	3	B
W161	F178		B
W366	F178		B
W327	F165	2	C
W207	F003		C
W305	F084	7	D
W153	F018		D
W102	F022		D
W105	F022		D
W106	F022		D
W108	F022		D
W141	F027		D
W049	FBBlandon	10	E
W132	F006		E
W309	F083		E
W201	F174		E
W290	F195		E
W299	F225		E
W330	F227		E
W043	FBBlandon		E
W044	FBBlandon		E
W046	FBBlandon		E
W359	F166	2	F
W325	F166		F

(For codes see Table S1).

Considering individual farms as separate, independent entities with individual compositions of genotypes, the average number of effective alleles present within all trees sampled at a single farm was 3.38 (range 1.0–5.4). Private alleles [9] occurred within only ten trees from seven farms (Table 2), including 8 of the 15 SSR loci investigated. The degree of expected heterozygosity (He) averaged over all 45 sites and 15 SSR loci was 0.476 (range 0–0.688). This is equivalent to an average of almost 50% (47.6%) of all loci being heterozygous. The rate of fixed loci was less than 30%, indicating a moderate degree of inbreeding at the current state.

**Table 2:** Private alleles at the farm level for 15 SSR loci across all 317 cacao trees sampled in Waslala, Nicaragua.

Sample (Tree)	Farm	Locus
W187	F005	mTcCIR24–193
W158	F018	mTcCIR33–350
W104	F022	mTcCIR26–281
W265	F029	mTcCIR7–147
W264	F029	mTcCIR11–307
W264	F029	mTcCIR18–346
W264	F029	mTcCIR33–273
W264	F029	mTcCIR37–144
W221	F143	mTcCIR22–291
W224	F143	mTcCIR22–291
W328	F165	mTcCIR37–186
W11	FERPozolera	mTcCIR18–333
W11	FERPozolera	mTcCIR18–343

### Estimation of the genetic diversity among farms

**Figure 1:**
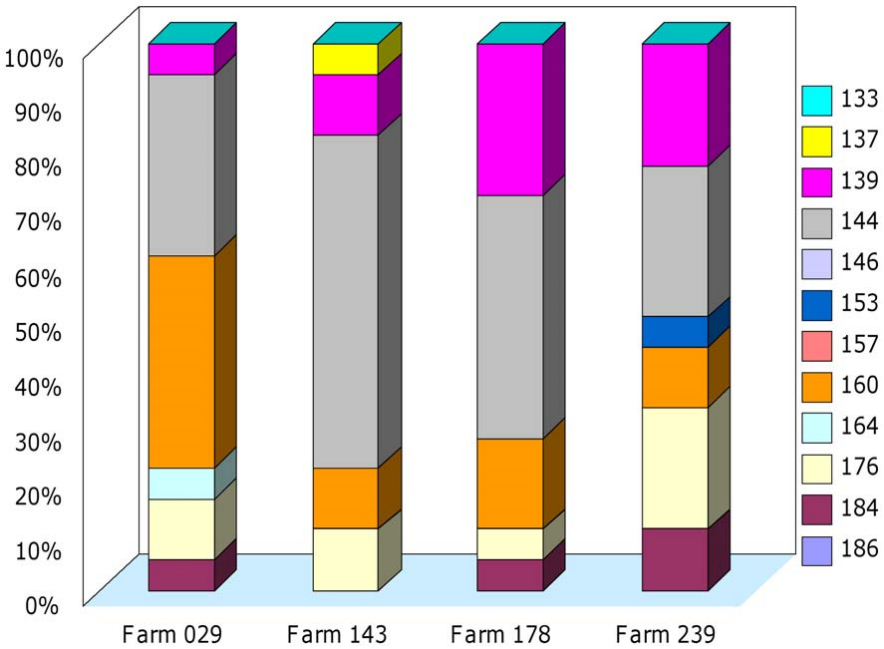
Frequency of SSR alleles (marker mTcCIR37) at four farms with samples from nine trees. A farm is represented by a single column showing cumulative frequencies of individual alleles. Alleles are indicated by their size in base pairs. Farms are labelled by their PCC numbers (also see Acknowledgement).

The degree of genetic diversity was calculated as the percentage of significant differences in all pairwise comparisons among farms, for every SSR locus in separate. Of a total of 14,864 comparisons by the G test using Shannon's mutual information index (^S^H_UA_) as implemented in GenAlEx [10], 39.4% were significant. This can be interpreted as showing considerable among-farm differences in frequency and composition of alleles at the 15 loci under study. The existence of large among-farm differences is further supported by the large differences in the frequencies of individual alleles by farm (e.g. Figure 1).

### Tracing the population structure across all cacao plantings in Waslala

Several simulations were performed in the program *Structure* [11] on all individuals and markers with and without consideration of the individual farms, as a factor contributing to the distribution of ‘farm subpopulations’ (LOCPRIORS option on or off, respectively). Simulations for up to K = 20 clusters were made. Each cluster was considered to represent one distinct group of ancestral genetic backgrounds that are referred to in this paper as a ‘genotype spectrum’ or lineage (known as ‘formenkreis’ in German). In contrast, a genotype as represented by a single individual can be made up entirely of just one genotype spectrum, or from parts of several such genotype spectra.

**Figure 2:**
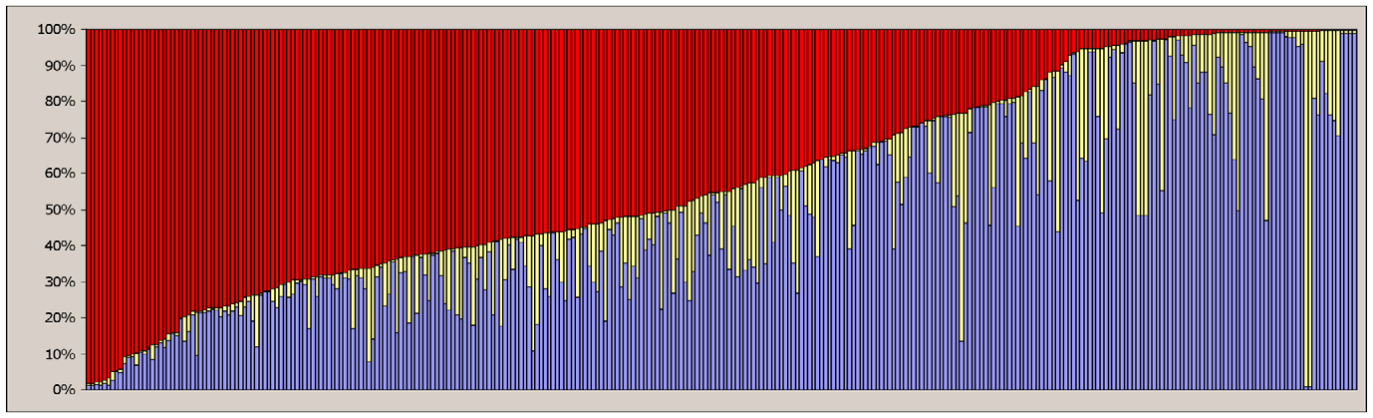
Bayesian clustering of cacao trees in Waslala. Best fit was achieved with three clusters representing three hypothetical founder genotype spectra, Blue, Red, and Yellow, with varying degrees of admixture within single individuals. A single column represents one of 317 individuals, with its proportions of the genetic lineages B, Y, and R. The two trees from forest remnants, W041 and W042, representing the ancient Criollo genotype spectrum, are indicated by the two entirely yellow columns.

The most probable number of populations (genotype spectrum clusters) was 3, as determined by a graphical method [12] as well as by the method applying Bayes' rule [13]. The partitioning of individuals across the three clusters was stable both with and without taking into consideration the location (farm). These three groups of genotype spectra, were denominated Blue, Red, and Yellow (B, R, and Y), for further investigation. Individuals within any of the three genotype groups contained different degrees of admixture from one or both of the other lineages (Figure 2). The Y group consisted of only three trees, namely the two FBBSB orphan trees from the forest (W041 and W042), and tree W357 from farm F204 (for identities, see supplementary Table S1). Tree W357 included a 14% admixture with components from the B lineage, and 23% from the R lineage. Another 12 trees, labelled as the BY admixture group, consisted of 30–50% Y, 30–50% B, and up to 20% R shared genotype spectra. There was also a BR admixture group of inferred genotype spectra (27–66% B, 33–65% R, 0–34% Y) consisting of 81 trees. A further 107 trees possessed a majority of B lineage components (39–99% B, 0–33% R, 0–32% Y), and 114 samples were mainly R (0–33% B, 42–99% R, 0–32% Y). Subsets of samples corresponding to the B or R clusters defined in this way were subjected to clustering simulations in *Structure*, but all attempts to detect sub-clusters within the B or the R genotype spectrum failed, and no further separation by the genotype spectrum was applicable within this set of data.

**Figure 3:**
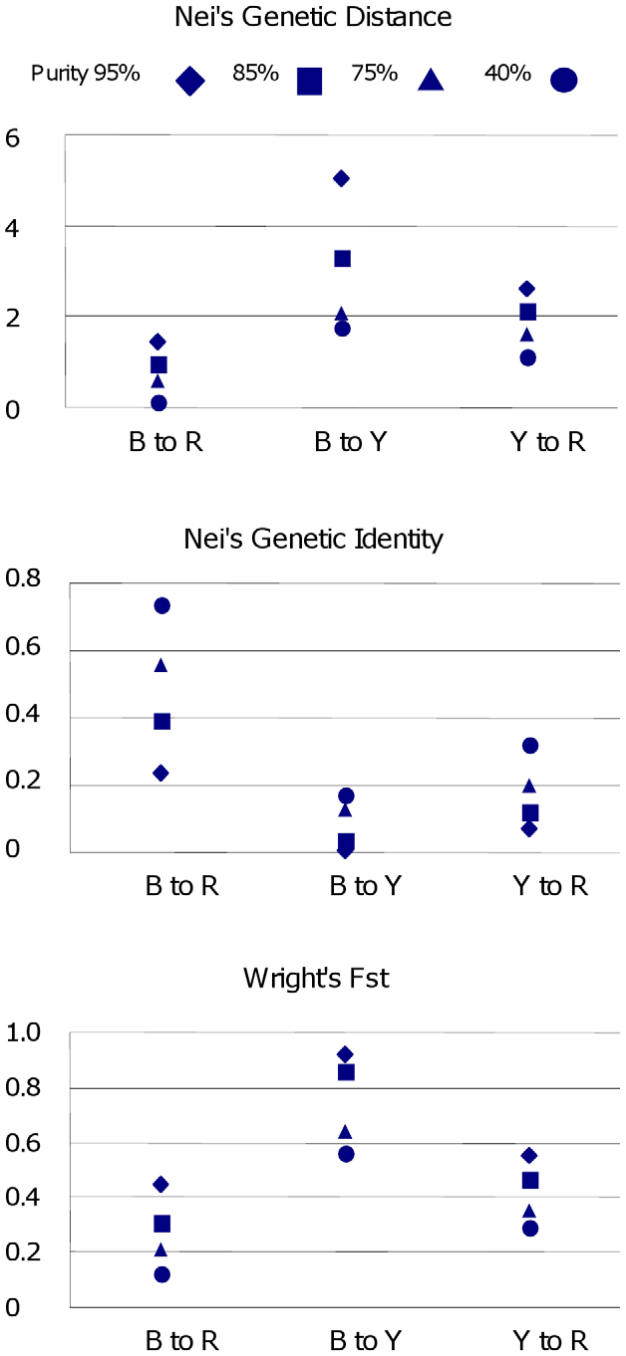
Nei's Genetic Distance and Genetic Identity, and Wright's Fst. Genetic distances between B (Blue), R (Red), and Y (Yellow) genotype spectra comprising groups of cacao trees whose admixed genotypes have certain minimum degrees of purity of the corresponding genotype spectrum (complementary to the maximum degrees of admixture with other genotype spectra, as shown in Figure 2). The increase or reduction of the parameter values throughout different degrees of purity in alignment support the clustering results shown in Figure 2.

The average genetic distance between the genotype spectra B, R, and Y was estimated by Nei's Genetic Distance and Genetic Identity and Wright's Fst as implemented in Genalex. Groups BR and BY with large admixtures were excluded. The results are presented in Figure 3. The closest related groups were B and R, with a Genetic Distance of 0.303, corresponding to a Genetic Identity of 73.8% and an Fst of 0.121. The Y group was most distant (Genetic Distance; 1.743 to group B and 1.141 to R), although this result must be taken with caution due to the small sample size of Y. The indices of relatedness were also calculated on reduced sets of samples restricting the portion of admixture genotypes. Allowing a minimum of 75, 85, or 95% presence of the B, Y, or R genotype spectrum (by removing samples with more than 25, 15, or 5% admixture, correspondingly), Genetic Distance increased and Genetic Identity shrunk as expected (Figure 3). This indicates that the clustering in the *Structure* program was successful in the detection of distinct genotype spectra.

The allelic diversity is largest in the R genotype spectrum group, followed by B, whereas the three samples representing the Y group possess very few different alleles per marker locus (Table 3). In fact, the two pure Y trees, W041 and W042, have perfectly matching alleles. Lineage R is also separated from B by having a larger number of private alleles. With increasing purity, i.e., virtually selecting for higher percentages of the prospected founder genotype spectra, the allelic diversity and expected heterozygosity decline, and the numbers of private alleles increase (Table 3).

**Table 3:** Allelic frequencies and parameters of clustered SSR multilocus genotypes among cacao trees in Waslala, excluding trees with extremely admixed (<39% purity) genotypes.

Minimum purity^1^	No. different alleles (Na)			
(15 loci)	n	B^2^	Y	R
39%	224	5.067	1.600	7.133
75%	116	4.400	1.600	6.267
85%	70	2.867	1.000	5.333
95%	32	1.533	1.000	3.600

	No. alleles with min. 5% frequency (Na> = 0.05)			
39%	224	2.733	1.600	4.867
75%	116	1.867	1.600	4.800
85%	70	1.467	1.000	3.933
95%	32	1.200	1.000	3.600

	No. effective alleles (Ne)			
39%	224	1.593	1.246	4.060
75%	116	1.379	1.246	3.778
85%	70	1.239	1.000	3.297
95%	32	1.159	1.000	2.273

	No. private alleles			
39%	224	0.267	0.000	2.267
75%	116	0.333	0.000	2.533
85%	70	0.400	0.067	3.333
95%	32	0.667	0.533	2.667

	Unbiased expected heterozygosity (UHe)			
39%	224	0.344	0.196	0.723
75%	116	0.246	0.196	0.711
85%	70	0.154	0.000	0.686
95%	32	0.094	0.000	0.574

	Shannon's Information Index (I)			
39%	224	0.693	0.268	1.523
75%	116	0.505	0.268	1.441
85%	70	0.297	0.000	1.326
95%	32	0.153	0.000	0.955

1 Increased purity (complementary to reduced admixture) simulates increased strength of selection for pure genotype spectra.

2 Genotype cluster, B; Blue, Y; Yellow, R; Red inferred founder genotype spectra.

Analysis of molecular variance (AMOVA as implemented in GenAlEx) on the B and R genotype clusters (assuming they represent founder genotype spectra) revealed 65% variation within and 35% among these clusters, and a Φ_PT_ value of 0.354 (P<0.001). A relatively small among-cluster variation was expected due to the fact that both lineages share the same alleles and possess large Genetic Identity values.

### Distribution of the three prospected founder genotype spectra at farm level

**Figure 4:**
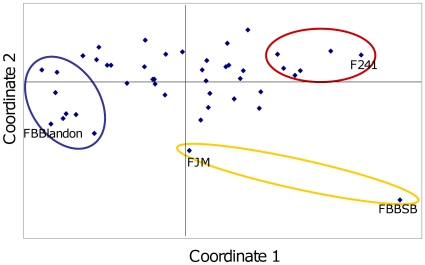
Principal Coordinates Analysis (PCA) on mean Shannon (sHua) values for pairwise farm comparisons. Plot of the first two main PCA axes. Comparisons included 15 SSR loci and 45 sites in Waslala, Nicaragua, represented by 317 cacao trees (first axis 39.9% and second axis 21.8% of total information). Circles indicate sites and farms with large portions (>75%) of the Blue, Red, and Yellow genotype spectra. Sites with largest shares of the genotype spectra are indicated by their code (compare with Figures 2 and 4, and Table S1).

**Figure 5:**
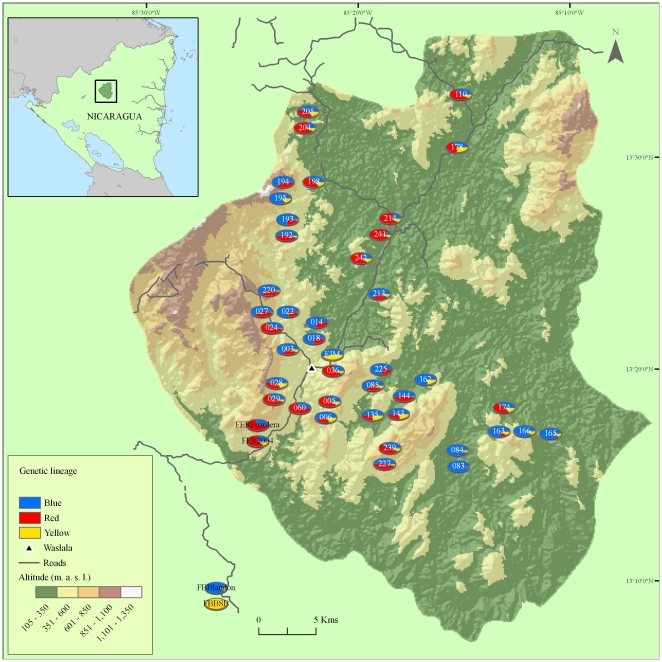
Map of Waslala municipality in central northern Nicaragua. Pie diagrams represent individual smallholder farms and the shares of putative founder genotype spectra, B, R, and Y, totalled over all cacao trees sampled.

As a measure of relatedness between different farms by genotype spectra composition, the average genetic distance over all 15 marker loci quantified by Shannon's index was applied. The results are summarized via principal coordinates analysis in Figure 4. Except FBBSB, the two orphan trees W041 and W042 near the forest, most farms were not well separated from each other by this method. This reflects the genetic composition of farms; with every farm having trees possessing genotypes of various states of admixture, considering the lineages as detected by the *Structure* program. That is illustrated by the pie diagrams on the map of Waslala municipality (Figure 5), each pie plot representing the proportion of the three genotype lineages contributing to an individual farm. The majority of farms are represented by tree genotypes made up of two (B and R) or three (B, R, and Y) lineages. Only a few farms consist of nearly exclusively B genotype spectrum partitions, and only the closely spaced south-eastern plantings F083 and F084, both owned by the same single farmer, contain nearly pure B lineage trees (Figure 5).

### Association among geographic characteristics of the sample sites, genotype spectra and geographic features

Pairs of the 5 continuous variables; distance to the road, altitude (m above sea level), elevation relative to nearest road (calculated as the difference between the altitude of the sample tree and that of the nearest point of the road), mean annual precipitation, and mean annual temperature, were subjected to correlation and regression analyses in a descriptive approach (Table 4). There was a small but highly significant correlation between the trees' distance to the road, and the elevation relative to the road, altitude, and temperature. A stronger correlation (R = 0.721) existed for distance to the road and mean annual precipitation. The elevation relative to the nearest road and absolute altitude were highly positively correlated (R = 0.799), meaning that trees at higher locations frequently grow on steep hills high above the neighboring roads. Consequently, the negative correlation of elevation relative to the road, and temperature, reflects the expected negative correlation between altitude and mean annual temperature (R = 0.946). This data (Table 4) also suggests that in this location, although mean annual precipitation tends to increase with increasing elevation as expected, some areas at low elevation receive much precipitation which may produce a cooling effect.

**Table 4:** Pairwise comparisons of climatic and geographic data for the locations of 295 sampled cacao trees representing the Blue, Red, and Blue-Red lineage clusters.

	Elevation relative to road	Annual precipitation	Altitude	Average annual temperature
Distance to road	−28.4***	72.1***	−25.6***	20.2***
Elevation relative to road		−13.5*	79.9***	−78.7***
Annual precipitation			12.1*	−15.7**
Altitude				−94.6***

Coefficients of correlation (in percent) and levels of significance (as determined by F tests in regression analyses) are shown.

*; P<0.05,

**; P<0.01,

***; P<0.001.

The discrete genotype spectra were used as a factor to compare geographical and climatic characteristics that they may be preferentially associated with (Table 5), in an exploratory approach. To avoid sampling bias due to grossly differing sample sizes, the under-represented groups Y (3 individuals) and BY (12 individuals) were excluded from these analyses. There were well-supported associations of individual genotype groups with the geographic distance to the nearest road, and mean annual precipitation (Table 5). The B genotype spectrum occurred more frequently at locations far from main roads (average 4.5 km) and the R and BR groups were frequently found nearer to roads (average 2.0–2.5 km). The group B was found in areas receiving the highest mean annual precipitation (2452 mm), whereas R and BR were not distinguishable in areas of 2409 mm mean annual rainfall. The elevation of R genotype spectrum trees above the nearest road was marginally but significantly above average. It is worth noting that replication, i.e., the individual trees at their given locations, also made a significant contribution to the total variance.

**Table 5:** Summary results of general linear models for analysis of variance of climatic and geographic factors for three abundant, inferred cacao genotype spectra in Waslala municipality.

Factor	Genotype spectrum P (F test)	Replication P (F test)	Multiple means comparison	Corresponding mean values
Distance to road	***	**	B BR R	4492 2534 1905 m
Elevation relative to road	*	-	R BR B	96 51 34 m
Annual precipitation	**	*	B BR R	2452 2411 2407 mm
Altitude	-	-	B BR R	428 438 458 m
Average temperature	-	-	B BR R	23.5 23.4 23.4°C

Levels of significance of dependent variables Genotype spectrum and Replication (representing individual trees within a genotype, used for calculation of the error term). Multiple means comparisons were made with the Waller-Duncan function in SAS-GLM; items connected by an underscore are not significantly different.

Number of samples included by genotype cluster; 107 B, 111 R, 77 BR.

*; P<0.05,

**; P<0.01,

***; P<0.001; –; (not significant).

In summary, genotype spectrum B occurred more frequently further from the road than the R genotype spectrum. Genotype spectrum B is more frequent at lower elevations with higher mean annual rainfall, whereas R occurs preferentially at higher elevations with lower mean annual rainfall, but R is more frequent than B higher above the closest road. This could be interpreted in the way that the R lineage is found preferentially in the mountainous part of Waslala municipality, where it is planted on slopes that steeply descend from the roads. The map (Figure 5) supports this notion. This also means that in the higher elevations (the mountainous south-west), the farms are located higher above the roads than in the lowlands. These higher altitudes with slightly increased mean annual rainfall experience lower temperatures, as is suggested by the strong, negative correlation (Table 4).

The two orphan trees, FBBSB, representing the pure Y genotype lineage, are located at an average altitude of 373 m at a relatively dry area (mean annual precipitation; 2333 mm; within the lower one sixth of the range for all trees sampled), where it is relatively warm (mean annual temperature 23.8°C; compared to the maximum temperature for all sampled farms being 24.9°C). Similarly, the 12 trees representing the BY group all grew in low, relatively dry and warm places (average for this group; 266 m elevation, 2396 mm mean annual rainfall, 24.5°C mean annual temperature).

**Figure 6:**
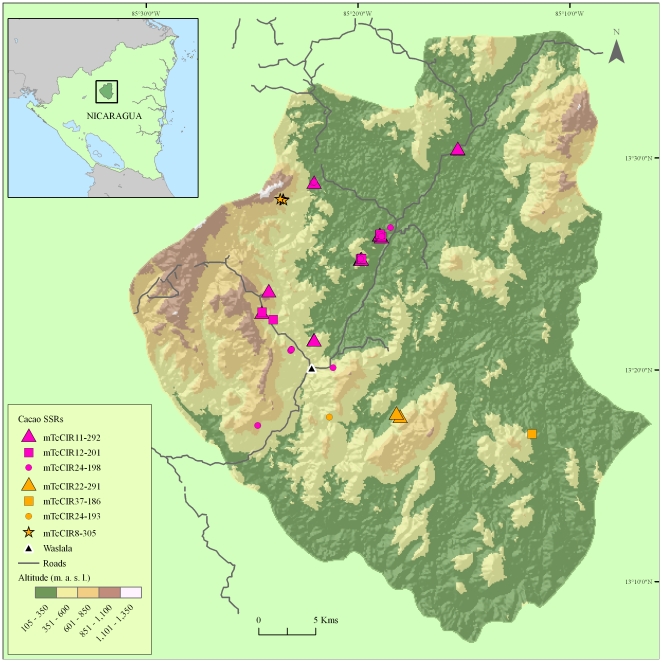
Map of trees possessing alleles confined to zones relative to the roads. Pink; alleles unique to areas close to the road (0–2 km; shared among 44 trees). Amber; alleles occurring far from the road (2–15 km; 7 trees).

Examination of the spatial distribution revealed that several single SSR alleles occur most frequently or exclusively in locations close to the main road (Figure 6). A total of 17 alleles are unique to a buffer zone of 2 km either side of the roads. As an example, allele mTcCIR292 occurs 18 times exclusively in these farms. In contrast, only four alleles were found uniquely in the area 2–15 km away from the nearest road. The number of effective alleles is also higher close to the road (3.2 within the 2 km corridor, relative to 2.36 further away; with the degree of expected heterozygosity, He, being 0.652 vs. 0.548, respectively). These increased levels of allelic diversity nearer to the roads suggest possibly more intense introduction of genetic materials along access roads. There is, however, a possible bias in sampling frequency (206 trees near, and 104 far from, the roads) that could interfere with part of these differences.

### Genetic lineage and fruit type

Assignment to one of three morphological fruit types, Acriollado, Común, and Híbrido, was achieved for 250 trees. Although all types were presented in all different locations, their ratios were not equal across the genetic lineages. The Común type was confined to the B lineage, except for a single individual in the R group (Table 6). For the two large groups B and R, whose members possess at least a two thirds share of the Blue and Red genotype spectra, respectively, the ratios of the frequent Acriollado and Híbrido trees were checked with the Chi-square test for goodness-of-fit. The B group had 34 trees assigned to the Acriollado type, and the R group had 8 of these. In total, in the Acriollado and Híbrido types, 85 B and 80 R individuals were recorded, therefore, 42.5 (85/2) B and 40 (80/2) R trees were expected to be encountered with the assumption of unbiased distribution of genetic lineages across the two fruit types (Table 6). Testing the observed frequencies of 34∶8, B∶R individuals to fit the expected ratio of 42.5∶40, revealed an unequal distribution or departure from homogeneity (Chi square = 14.28, P<0.001; ***). This allows the conclusion that the Acriollado morphotype is highly significantly underrepresented in the R lineage, and overrepresented in the B lineage. This was the most pronounced biased distribution found; with 81% of the Acriollado type within all B and R samples being present among the B lineage trees. Likewise, the Híbrido fruit type, albeit outnumbering the other varieties, was cumulated at the 5% level of marginal significance to the R genotype spectrum. Testing the observed frequency of 34∶51 Acriollado∶Híbrido individuals within the B genotype spectrum, and 8∶72 within R, to fit the expected ratio of 21∶61.5, revealed a similar bias at P<0.01 (**) in both comparisons.

**Table 6:** Frequencies of morphological fruit types relative to inferred genotype spectrum group.

All trees identified							
Fruit type\genotype	B^1^	BY	BR	R			Total
Acriollado	34	2	22	8			66
Común	9	0	0	1			10
Híbrido	51	8	43	72			174
Total	94	10	65	81			250

B and R groups only^2^	B	R	Total	observed	expected	Chi squ	P
Acriollado	34	8	42	34∶8	42.5∶40	14.28	***
Híbrido	51	72	123	51∶72	42.5∶40	5.27	*
Total	85	80					
observed	34∶51	8∶72					
expected	21∶61.5	21∶61.5					
Chi square	9.47	10.06					
P	**	**					

1 For codes of genotype spectrum (or founder genetic lineage) clusters, compare legend of Table 3. BY; cluster containing individuals with admixed Blue-Yellow, BR; Blue-Red, genotypes at equal proportions.

2 Bottom section; fitness-to-homogeneity tests of frequencies across genetic lineage and fruit types showing observed and expected numbers of individuals, Chi square value, and corresponding probability level, P (*; P<0.05, **; P<0.01, ***; P<0.001).

## Discussion

The genetic structure of smallholder cacao plantings in Waslala was investigated. This is an economically significant Nicaraguan area of production, where this crop has been grown since 1961. The majority of these cacao plantings appear to possess a large diversity of tree genotypes that seems to originate from a limited number of genotype spectra. Notwithstanding, the differences in allele and genotype composition at the farm level are important.

### Markers used and allelic diversity

The 15 microsatellite loci sampled in this study are dispersed across nine of the ten linkage groups (chromosomes) of *Theobroma cacao*. These loci were selected as robust, informative markers for cacao and have been characterized in detail [14]. The 15 markers have been widely used to assess the genetic diversity and redundancy among new cacao collections and within clonal collections held at genebanks [1], [3], [5], [15]. Therefore, these markers were considered appropriate to assess the cacao genepool present at the municipalities of Waslala/Rancho Grande, Nicaragua. The markers are anonymous and unlikely to target specific expressed genes, therefore they can be considered as neutral, i.e. not under selection and thus are unbiased markers for this investigation of population structure.

To assess the allelic diversity in Waslala, the total number of alleles, and private alleles, can be used. The 116 individual alleles found within the samples are almost exactly one-half of the number of 231 alleles observed for the same loci among 548 accessions with distinct genotypes that were sampled by Zhang et al. [15] at the live cacao genebank in CATIE, Costa Rica. This means that the allelic richness in Waslala of 7.73 alleles per microsatellite locus, is approximately 50% of the richness within the CATIE collections that have 15.4 alleles per locus. The collection of the USDA-ARS Tropical Agricultural Research Station at Mayaguez, Puerto Rico, holds at the same SSR loci in total 132 alleles with 8.8 alleles per locus [16], being comparable to Waslala, although actual differences in the individual alleles are likely to exist. The level of allelic richness in Waslala is also comparable to that of a collection of semi-natural cacao from the upper Amazon, held at Universidad Nacional Agraria de la Selva, Tingo Maria, Peru [17], with allelic richness levels comparable to that of the USDA-ARS Mayaguez collection [18]. A subgroup of Ecuadorian cacao collections recognized as being the genetically narrow ‘Refractario’, had in total 63 alleles and 4.2 alleles per locus [19]. Again, the identities of the alleles may be different although the same microsatellite loci were investigated.

### Cacao population structure across plantings in Waslala

Of the 13 private alleles detected by the rarefaction method (Table 2), 8 are dispersed among only 7 trees from three farms. This supports the notion of the wide dispersal of a comparatively small set of common alleles across Waslala, although there is much diversity at the genotype level (a specific combination of alleles at all loci). Evidence for this arises from the occurrence of only a few highly similar SSR genotypes. There are only 7 groups of trees with matching genotypes (Table 1), pointing towards sufficient genetic recombination, probably achieved through planned crosses. The small number of matching genotypes also indicates that during the sampling, trees of clonal origin were successfully omitted. The main method of tree propagation in Waslala is by seed, although in recent years, grafting of scions onto established rootstocks of trees that are cut due to low productivity, has become an alternative method.

The experimental station and germplasm distribution unit in Nicaragua, El Recreo, receives cacao germplasm from CATIE, and apparently, seed from crosses at El Recreo were distributed to Nicaragua's production zones including Waslala. During 1991–96, considerable dispersal of seed from controlled Trinitario×Forastero crosses and from clonal propagation of superior Trinitario genebank accessions was recorded in Waslala (S. Thienhaus, FADCANIC, Centro Agroforestal Sostenible, Wawashang, Nicaragua, 2010, pers. comm.), and the Cacaonica cooperative was involved in the distribution of this germplasm to farms sampled in this study. Nonetheless, the data on alleles and genotypes shows that the material used may have been selected from certain parts of the genotype spectra available in cacao.

The considerable differences were observed in the frequency and on-farm composition of genotypes across farms, as witnessed by the spatial distribution of genotypes with widely differing degrees of lineage admixture (Figure 5). This may reflect seed trade activities of the past. Nonetheless, neither differentiation-based diversity (principal coordinates analysis on mean Shannon values, Figure 4), nor probabilistic inference of population structure [20] revealed any indication of more than three distinct genotype spectra within all samples from Waslala. Likely causes for this include the preference by farmers for only a few sources of genetic material for unknown reasons, newly introduced trees of <20 years of age may not yet be among the high-yielding trees and were thus not sampled, or the parents used for the crosses were closely related. The inference of population structure applied here can only give information on the number of genotype spectra that are discernible in the existing data set. However, it cannot assess the absolute magnitude of diversity any of these single genotype spectra consists of. Likewise, at this stage it is problematic to trace any individual donors of the B and R genotype spectra due to the large number of choices that are available at the genebanks (e.g. SSR fingerprints of clonal accessions offered by the International Cocoa Germplasm Database; www.icgd.reading.ac.uk/index.php). This can be achieved by integrating the current data on the Nicaraguan populations with information on particular parental material that may have contributed to this genepool.

### Origin of the Y genotype

Of the inferred three founder genotype spectra, two, B and R, were frequent and widespread, whereas only the two cacao trees from the forest, W041 and W042 represented the pure, non-admixed Y lineage. Several instances point toward the assumption that the Y trees may indeed represent the ancient Criollo lineage. The two forest trees were growing in a wild state, and appeared significantly older than all the managed plantation trees. Farmers do not harvest fruit from such forest trees because of their low yield and small, unpigmented seed. Criollo is known to possess extremely small allelic diversity, small unpigmented seed, and exhibit low yields. The majority of Criollo trees were killed by an unknown incident in 1727 [21], and only a few plants apparently escaped by chance, with rare trees to be found at sheltered sites near ancient settlement places in this Central American region [2], [3]. However, confirmation of the two orphan trees being Criollo will require additional comparative studies.

### Potential identity of the B and R genotypes and their spatial distribution patterns

The B and R lineages are present predominantly in admixed states (Figure 2), and residues of the Y lineage were detected by the probabilistic clustering method within a minority of the BR hybrids. Y-admixture could mean hybridization with Y representatives in the past, but it could also mean that intercrosses among introduced BY hybrids could have split the putative Y lineage into the presently observed fragmentary levels. Such parental hybrids could be Trinitario accessions which are hybrids of Mesoamerican (Criollo) and Amazonian (Forastero) cacaos [3], [4], [5]. Whether the B or the R lineages, or both, could represent Trinitario cannot be discerned with the data available. The fact that no clear R-Y hybrids were found among the 317 samples could be the result of insufficient time for this hybridization to take place. It also suggests that R may represent most recent introductions that have been intentionally hybridized with B, for example in the crossing and propagation programs conducted in the early 1990s at the Cacaonica cooperative and by other organizations. The hypothesis that the R lineage was only recently introduced is shown by its preferential distribution near to main access roads and around the town of Waslala itself (Figures 5 and 6).

The B lineage is more widespread in plantings situated relatively further from main roads. This could reflect farmers' habits of distributing their plants or, they could be a remnant of two successive periods of introduction, the B lineage being older. It is, however, somewhat remarkable that the R genotypes have not found a wider distribution within the purported 15–30 years since their likely arrival, as the maximum distances from the main roads within the municipality rarely exceed 15 km (Figure 5).

Microclimate-driven spatial distributions of individual genotypes within wild plant (including grass and tree) populations have been observed. In nature, subtle differences of shading [22], temperature and precipitation variation [23], or precipitation and soil alkalinity [24], are sufficient to strongly influence population structure. At the relatively young plantings found in Waslala, that vary from 13 years old to maximum of 49 years old, single trees are quickly replaced when they are unproductive, affected by diseases, or when more promising planting material become available. Under these circumstances, and because the majority of locally available material belong to only two basic genotype spectra, it cannot be excluded at present that microclimatic variations, in particular precipitation, may be a factor that partially determines the spatial distribution of these genotypes, alongside management practices. Again, clarity can only be obtained through additional experiments.

Remarkably, the Y lineage putatively representing the ancient Criollo type has a narrow distribution in an area that experiences relatively low annual precipitation and relatively higher mean annual temperatures. This may point to the preferred environmental conditions that facilitate the survival of this lineage under unmanaged conditions, and may help to elucidate the nature of the unknown incident that wiped out the Criollo crop in 1727 [21]. However, these findings must be treated with caution due to the small number of Y individuals.

### Distribution of fruit types

Despite the great variability of morphological characteristics, the distribution of types identified by fruit shape, seed color and size (Acriollado and Común varieties) and in addition, to a limited extent the technology of production (for the Híbrido type), was unequal across the three inferred genotypes B, Y, and R. The B genotype contained nine of the ten Común-type trees distinguished by their melon shaped fruits. Among the two main genetic lineages, B and R, B represented most of the Acriollado type trees (Table 6). The Híbrido type is the only vernacular ‘variety’ that is applied to trees based on a mix of categories; fruit morphology and recorded technique of their production by controlled crosses. Accordingly, trees recognized as Híbrido occurred in all inferred genetic lineages at high frequencies, although R, the lineage with the largest distribution along main roads and more influenced by new introductions, had slightly more Híbrido individuals than B, at the marginal significance level of P<0.05. Therefore, fruit and seed morphology are, at least in part, genetically determined, and can be selected for by visual examination. Exploring the features that lead to the identification of vernacular varieties as is demonstrated with Híbrido trees, is recommended. However, as the designation to this type is based on a mixture of natural and technical criteria, its usefulness is limited.

In conclusion, the multilocus genotypes as detected by the 15 microsatellite markers can be used directly to denominate and recognize individual cacao trees and farms. This opens a means to select and breed for further enhancement of the crop and diversification of cocoa quality, both within the entire area and at the farm level. Of the two scenarios for future breeding, enhancement using the existing germplasm, or hybridization with superior imported material, the latter could likely disturb the already established and valued site-specific cocoa quality based on existing alleles and genotypes. Multi-year measurements of the culinary quality of cocoa and chocolate from the sampled trees are under way, and if these experiments reveal distinct features of the lineages, this could open up opportunities for breeding and selecting genotypes conferring elite quality.

## Materials and Methods

Forty four cacao plantings in smallholder farms were selected to represent 14 climatic zones within the municipality of Waslala, Nicaragua. Two naturally occurring orphan cacao trees remaining from recently cleared forest were also included. This group is referred to as derived from “farm FBBSB”.

A total of 315 trees identified were selected as consistently high yielding by their owners, and two low-yielding FBBSB trees, on average 7 trees per location (range 2–20). Eight locations were represented by less than 5 trees. High yield was defined as the stable production of many fruits year-round. This ‘high yield’ of individual trees as observed by the farmer may depend on the degree of stylar self-compatibility, distance from neighboring cacao and shade trees, and degree of fertilization, rather than on the genotype, and the principle of random sampling was therefore adhered to. Care was taken to sample non-grafted seedlings. New, fully expanded adult leaves were dried on silicagel in sealed plastic bags and shipped and stored at room temperature until use. Total genomic DNA was extracted from dry leaf tissue with the Dneasy Plant Mini Kit (Qiagen) according to the manufacturer's protocol.

Three types, mainly defined by morphological characteristics of the fruit (pod) and seed (beans) were identified. Acriollado has white beans, and pulp color and fruit shape with some resemblance to the original Criollo type. Común was used to describe trees producing fruits of one Forastero morphotype, namely Amelonado, possessing spherical pods similar in shape to honey melons (*Cucumis melo*). Finally, Híbrido was used to describe plants producing pods of intermediate shape and characteristics, as they occur frequently after hybridizing crosses of Forastero and Criollo. These pods often are elongated with pronounced acuminate tips and reduced seed size. The Híbrido classification was also applied to trees reported to be obtained from seed programs by the Nicaraguan genebank, El Recreo, or by the Honduran Foundation of Agricultural Research (FHIA), that are creating varietal hybrids through controlled crosses.

Primers for 15 simple sequence repeat (SSR or microsatellite) markers [25] specified in Table 7 were purchased from Sigma. For each marker, one of the primers was labelled with a fluorescent dye (FAM or HEX), and the PCR amplicon was separated on ABI Prism 3100 and ABI Prism 3130xl capillary sequencers to visualize the microsatellite alleles. The data generated in the Sequencher 4 software (Gene Codes Corp., Ann Arbor, USA) was analyzed with the aid of Genotyper, Peak Scanner 1 (ABI), or Genemapper programs. The individual alleles were labelled by the size in bases of their largest repeat. The PCR was replicated to up to five times to eliminate uncertainties. Together with newly shipped samples, previously analyzed control samples were included to provide the correct assignment of allele sizes. For each sampled tree, DNA was isolated once or twice. Trees were sampled during three years, from 2007 to 2009. For several trees, a second leaf was sampled in a different year.

**Table 7:** Cacao microsatellite (simple sequence repeat; SSR) primers [27] used to fingerprint trees from plantings in Waslala, Nicaragua, 2007–2009.

SSR code^1^	EMBL No	5′-Primer	3′-Primer	Chr	Size (bp)	AT °C
mTcCIR1	Y16883	GCAGGGCAGGCTCAGTGAAGCA	TGGGCAACCAGAAAACGAT	8	128–146	59
mTcCIR6	Y16980	TTCCCTCTAAACTACCCTAAAT	TAAAGCAAAGCAATCTAACATA	6	225–247	48
mTcCIR7	Y16981	ATGCGAATGACAACTGGT	GCTTTCAGTCCTTTGCTT	7	147–162	53
mTcCIR8	Y16982	CTAGTTTCCCATTTACCA	TCCTCAGCATTTTCTTTC	9	286–305	50
mTcCIR11	Y16985	TTTGGTGATTATTAGCAG	GATTCGATTTGATGTGAG	2	287–337	48
mTcCIR12	Y16986	TCTGACCCCAAACCTGTA	ATTCCAGTTAAAGCACAT	4	186–220	55
mTcCIR15	Y16988	CAGCCGCCTCTTGTTAG	TATTTGGGATTCTTGATG	1	231–257	50
mTcCIR18	Y16991	GATAGCTAAGGGGATTGAGGA	GGTAATTCAATCATTTGAGGATA	4	330–354	53
mTcCIR22	Y16995	ATTCTCGCAAAAACTTAG	GATGGAAGGAGTGTAAATAG	1	272–291	48
mTcCIR24	Y16996	TTTGGGGTGATTTCTTCTGA	TCTGTCTCGTCTTTTGGTGA	9	185–202	51
mTcCIR26	Y16998	GCATTCATCAATACATTC	GCACTCAAAGTTCATACTAC	8	281–306	46
mTcCIR33	AJ271826	TGGGTTGAAGATTTGGT	CAACAATGAAAATAGGCA	4	271–350	53
mTcCIR37	AJ271942	CTGGGTGCTGATAGATAA	AATACCCTCCACACAAAT	10	133–186	50
mTcCIR40	AJ271943	AATCCGACAGTCTTTAATC	CCTAGGCCAGAGAATTGA	3	258–294	51
mTcCIR60	AJ271958	CGCTACTAACAAACATCAAA	AGAGCAACCATCACTAATCA	2	186–211	53

1 The code of the SSR and corresponding EMBL accession number, PCR primers, number of the cacao chromosome (Chr), fragment size (Size), and PCR annealing temperature (AT) are indicated.

Basic parameters on the samples' genetic composition and allele frequencies were calculated using the GenAlEx application [10] in Microsoft Excel. Principal coordinates analysis (PCA) and analysis of molecular variance were also performed in GenAlEx. For PCA, the mean Shannon mutual information indices (sHua) for pairwise farm comparisons were calculated as the fraction of Total Information index across each pair of populations, which were comprised of the weighted Allele Information indices of both populations in the pair, for each locus (compare www.anu.edu.au/BoZo/GenAlEx/new_version.php, GenAlEx Tut1, p. 35). The genotypes were further analyzed with Bayesian statistical methods in the program *Structure* [11] to attempt to trace the number and genetic composition of founder populations or kinships in Waslala cacao plantings. Settings for the simulations in *Structure* were 100,000 permutations during the burnin phase and 50,000 to 100,000 during simulations under a model allowing for genotype admixture.

Spatial climate data were extracted from Worldclim (www.worldclim.org). This database provides detailed information on climate characteristics at 1 km×1 km-resolutions, and its estimated tolerance of annual precipitation values is 10–25 mm for this part of Central America [8].

Geographic information system (GIS) analyses and maps were made with the DIVA-GIS software (www.diva-gis.org). Administrative and access information was based on maps by MARENA, the Nicaraguan Ministry of Environment and Natural Resources [26].

Planned potential associations among geographic and climate variables and inferred genotypes were tested by correlation and regression analyses in Excel or by general linear models in SAS (SAS Institute Inc., Cary, USA), of the type Y = βX+*e*, where X is the discrete genotype, *e* the error represented by the replication dependent variance, and Y the individual factor of influence, where appropriate. The individual trees within one genotype group were considered as replications for this genotype.

## Supporting Information

Table S1 List of the 317 cacao trees. Owner; farmer's name. Comarca; rural district. Lineage; inferred genotype spectrum. Climate zone; defined by average temperature and precipitation. CIR1–CIR60; SSR fingerprint. The two alleles at each SSR locus are listed in two columns within one row.
(XLS)
